# Medical Protective Association of New Orleans

**Published:** 1875-08

**Authors:** 


Medical Protective Association of New Orleans.—The physicians of New Orleans have formed an association for mutual aid and protection. So many and such overgrown abuses of the policy of the profession, in regard to its emoluments, have been allowed to encumber its practice in this city, that instances of actual thrift have become the exception; retrogression in business and in financial prosperity is the rule. The evils have become so pinching in their effects that the sufferers finally began to look to each other for counsel and support, and thus has resulted the organization of the "New Orleans Medical Protective Association."

What a wonderfully self-contradictory agent self-interest is. In prosperity its tendencies are centrifugal; when want or woe threaten they suddenly become centripetal. Now these reflections are simply in the way of the most innocent soliloquy, without the least breath of invective. I know, and every physician knows, that there is no part of the civilized world in which the medical profession is not freely permitted to determine and regulate for themselves all matters concerning their fees and emoluments. And this permission is sustained both by legal countenance and by the consent of communities among which they practice. The city of New Orleans is not only no exception to this statement, but affords an illustration of a community remarkably liberal to its learned professions, as long as liberality remained a possibility. The move made by our profession is a good one. After having committed the great error of wronging each other by accepting the salaries of heartless associations, whose pay for the services rendered was at the rate of from "five cents" to "twenty-five cents" per visit—after taking families year after year at ruinously cheap rates, lest some others of the brethren might supersede them; after competing with each other, more after the order of rival inns in a country village, than members of a dignified and honorable profession, at last the light bursts upon us, and we see how impolitic, improper and unseemly it is to tolerate selfish conduct in our ranks, or to fail to rebuke every exhibition of trickery tending to sink us to the level of petty, haggling traders.—*New Orleans Medical and Surgical Journal.*

